# Route flexibility is associated with headwind minimization in a long-distance migratory seabird

**DOI:** 10.1098/rspb.2024.2522

**Published:** 2025-04-02

**Authors:** Nathalie Kürten, Joe Wynn, Birgen Haest, Heiko Schmaljohann, Oscar Vedder, Jacob Gonzalez-Solis, Sandra Bouwhuis

**Affiliations:** ^1^ Institute of Avian Research, An der Vogelwarte 21, Wilhelmshaven 26386, Germany; ^2^ Swiss Ornithological Institute, Seerose 1, Sempach 6204, Switzerland; ^3^ Institute of Biology and Environmental Sciences, University of Oldenburg, Carl-von-Ossietzky-Straße 9-11, Oldenburg 26129, Germany; ^4^ Institut de Recerca de la Biodiversitat and Departamento Biologia Evolutiva, Ecologia i Ciències Ambientals, University of Barcelona, Av. Diagonal 643, Barcelona 08028, Spain

**Keywords:** movement ecology, bird migration, migratory behaviour, migration route consistency, wind effects, geolocation, nearest neighbour distance

## Abstract

Seasonal migration has evolved across taxa and encompasses a multitude of features, many of which vary between species, between and within populations, and even within individuals. One feature of migration that appears especially variable within individuals is the route taken to reach a destination, even when the destination itself is not variable at this level. To investigate why, we analysed the geolocator tracks describing 192 post-breeding migratory journeys of 84 common terns (*Sterna hirundo*), as well as 149 pre-breeding migratory journeys of 75 of these birds. We found little within-individual spatial consistency in migration routes across years, irrespective of season or sex. Instead, individuals departing during the same time window took similar migration routes, which, during pre-breeding migration, when birds predominantly encountered headwinds, were associated with minimized headwind exposure. We therefore suggest that the individual routes of this long-distance migratory seabird can be flexibly adjusted to environmental variation, which is likely to be adaptive.

## 1. Introduction

Animal movement is organized across a variety of spatial and temporal scales, with seasonal migration being one of the most spectacular examples. Seasonal migration encompasses a multitude of features, many of which show different levels of variation both between and within species, populations and individuals [1]. At the level of the individual, limited variability in migratory behaviour may be underpinned either by behavioural constraints that do not allow individuals to respond to dynamic cues [2] or by consistency being favoured because familiarity offers selective advantages (e.g. [3]). In a changing world, however, a limited ability to flexibly respond to varying rates of environmental change across the annual range may hamper adaptation (e.g. [4,5]) and has, indeed, been suggested to contribute to the rapid population declines observed in many migratory species (e.g. [5–7]). As such, determining the degree of individual consistency or flexibility in aspects of migratory behaviour, as well as how it relates to environmental variation, is of importance, not only when aiming to understand the evolution of the migratory phenotype, but also for assessing whether animals may be able to respond to future environmental change.

Technological advances have allowed biologists to track a large range of individual animals across their annual cycle, which in turn has revolutionized our understanding of year-round species distributions and individual-level migratory behaviour. Moreover, repeated tracking of subsets of individuals is increasingly facilitating the determination of the degree of individual consistency in migratory behaviour through space and time (for a review, see [8]). So far, within-individual consistency in the timing of migration and in the locations of the breeding and non-breeding residence areas appears substantial and widespread, while spatial consistency in the routes taken to reach these consistent residence areas has received less empirical attention (but see e.g. [9–11]). When quantified, however, route consistency appeared relatively low (e.g. [12–19], but see [20]), leading to the question why this is so.

To understand migration routes and their (in)consistency, we must, as also alluded to above, consider the sources of variation underpinning them. Among birds, migration routes are typically, but not universally [21], thought to be inherited genetically as a ‘clock and compass’ vector: a compass to encode direction and a clock to encode distance [22–24]. Such genetic information is thought to be supplemented with information gained through both asocial associative (‘trial and error’) learning [25–27] and social learning from experienced conspecifics [28,29], especially in long-lived species. Both in the case of a genetic basis to orientation and in the case of learning through experience, we might expect high levels of individual route consistency, which could be of considerable advantage if it would prevent individuals from entering potentially risky novel environments (e.g. [30]).

During migration, however, birds must deal with a highly dynamic environment in which conditions, especially wind, may change unpredictably through both space and time (e.g. [31]). Wind conditions are known to be a major driver shaping migration routes, at least across individuals (e.g. [32]), and long-distance migrants have been found to maximize wind efficiency rather than spatial efficiency (i.e. to minimize the headwind encountered rather than the total route length; e.g. [33,34]). With respect to within-individual consistency in migration routes, we might expect this to be low if individuals can flexibly adjust their routes to prevailing wind conditions to maximize their migratory efficiency (e.g. [35–37]) and/or if wind drift would, for example, lead to involuntary route changes [38]. Testing whether wind conditions indeed might explain variation in the (in)consistency of routes within individuals or across time will help us gain insight into one of the mechanisms underpinning individual ecological responses to changing environmental conditions [39].

Here, we report on a multi-year individual-based study in which we used light-level geolocators to track 192 post-breeding migratory journeys of 84 European common terns (*Sterna hirundo*), as well as 149 pre-breeding migratory journeys of 75 of these birds to test whether birds show within-individual spatial similarity in the migration routes taken across years. We also assessed whether birds leaving within the same time window (and hence experiencing similar environmental conditions) take similar migration routes, and if so, whether such among-individual temporal route similarity could be associated with minimization of the encounter of unfavourable wind conditions.

## 2. Methods

### (a) Study species and data collection

Common terns are Holarctic colonially breeding and long-distance migratory seabirds [40] that display high site fidelity to both their breeding site [41,42] and non-breeding residence area ([43], also see §3). To understand whether they display route consistency as well, or how wind conditions may affect the routes the birds take as they migrate to their set destinations, we studied common terns at a monospecific colony located at the Banter See in Wilhelmshaven at the German North Sea coast (53°30′40″ N, 08°06′20″ E), using archival light-level geolocators (Intigeo-C65, Migrate Technology, UK).

Between mid-May and early July 2016–2020, we caught 84 incubating common terns a total of 229 times with a drop trap, on average 16 ± 4.3 s.d. days after the first egg in their nest was laid. These birds were then equipped with a geolocator attached to the leg using a 10 mm aluminium ring [44]. The geolocators were set to sample ambient light intensity every minute, with the maximum light intensity being stored every 5 min (mode 10). The total mass of the geolocator, ring and glue was 1.6 g, which equalled 1.2% ± 0.1 s.d. of the body mass of the birds at capture (mean body mass: 129.1 g ± 8.1 s.d.). In the breeding seasons of 2017–2021, we re-trapped the 84 individuals equipped with a geolocator that returned (i.e. 86%) a total of 198 times and recovered 192 devices with light-level data (i.e. 97%). As such, we obtained data for 192 post-breeding migratory journeys performed by 84 individual birds (41 males and 43 females), as well as 149 pre-breeding migratory journeys performed by 75 of these birds.

### (b) Analysis of light-level geolocator data

To analyse the light-level data, we used the function ‘preprocessLight’ of the R package ‘BAStag’ [45] and a threshold of 1.5 to identify twilights. If visual inspection suggested extreme outliers (>30 min difference with the previous and subsequent twilight), we either adjusted the outlying twilight or excluded it (*ca* 1% and <1%, respectively; as calculated based on the light-level datasets of 10 randomly selected individuals).

All geolocators were calibrated ‘on-bird’ while the birds were at the Banter See colony (i.e. for multiple weeks). The start and end of the calibration periods were individually determined by visual inspection of the calibration slopes for sunsets and sunrises plotted by the ‘plot_slopes_by_location’ function of the R package ‘FLightR’ (v. 0.5.0 [46]). As long as the bird stays in the calibration location, these slopes vary very little [46], so the first calibration period ended with a visual detection of a change in the slope (i.e. when the bird was assumed to have departed). For geolocators that were still working at recapture, a second calibration period was set to start when the slope did not show variation anymore (i.e. when the bird had arrived).

For the subsequent movement analyses, we set the mean flight distance between two twilights to 1200 km, i.e. 24 h × 50 km h^−1^ [47], while allowing for ± 300 km, and ran the particle filter with 1 million particles to optimize the track of each individual and to minimize its uncertainty [48]. The uncertainty of location estimates obtained with ‘FLightR’ is approximately 250 km per location (dependent on shading caused by the species’ behaviour), which is substantially less than that of estimates obtained with more traditional threshold methods [46,49]. We deem this uncertainty to be unlikely to impact our analyses substantially, since all tracks are expected to be equally affected, common terns migrate a distance (5000–11 000 km; [43]) that is relatively large compared with the uncertainty in the location estimate, and our large sample size (*n* = 192 post-breeding and 149 pre-breeding migratory journeys) secures statistical power.

Finally, we applied the ‘stationary.migration.summary’ function of ‘FLightR’ with a ‘min.stay’ of 10 twilights (i.e. 5 days) and a ‘prob.cutoff’ set to 0.4 (for detailed testing and explanation of these settings, see [43]) to derive the longitude and latitude of each non-breeding residence area, as well as the dates of arrival to, and departure from, the breeding colony and each non-breeding residence area for each track from each bird. For 185 tracks from 81 individuals, we obtained a single non-breeding residence area, whereas for seven tracks of six individuals, we obtained two in at least one of the tracked years. The post- and pre-breeding migration routes were obtained from the daily location estimates between the departure dates from, and arrival dates to, the breeding colony and first and last non-breeding residence area, respectively.

### (c) General spatial and temporal consistency

We calculated the intra-individual repeatability for the post-breeding departure date from the breeding colony, post-breeding arrival date at the (first) non-breeding residence area, longitude of the (first) non-breeding residence area, latitude of the (first) non-breeding residence area, pre-breeding departure date from the (last) non-breeding residence area and pre-breeding arrival date at the breeding colony. Repeatability is statistically defined as the proportion of the total variation in a trait that can be attributed to differences between (compared to within) individuals, and for its calculation, we ran five separate models, each fitted with a normal error distribution, year and sex as categorical fixed effects, and individual identity as a random intercept using the ‘rptGaussian’ function of the R package ‘rptR’ [50]. We report the mean (±s.e.) repeatability estimates, as well as the 95% confidence intervals (CIs) obtained from 1000 bootstrap iterations, and *p*-values.

### (d) Route similarity within birds across years and among birds leaving at the same time

We did not test for within-year, among-season similarity of migration routes within individuals, because we found post-breeding migration routes to be vastly different from pre-breeding migration routes, excluding the option for similarity altogether (electronic supplementary material, figure S1). First, we therefore, compared season-specific pairs of individual tracks to assess how similar birds were to themselves across years as they travelled between their breeding colony and non-breeding residence area (i.e. season-specific within-individual route similarity). Second, we compared pairs of tracks of individuals leaving the breeding colony/non-breeding residence area within a short time window within the same year (here defined as the date of the onset of the focal track ±3 days, so a 7 day window (the smallest window returning sufficient sample size)) to assess what we term the ‘among-individual temporal route similarity’. To this end, we used the nearest neighbour distance (NND), a metric designed for this purpose [51], and commonly used (e.g. [9–11,52,53]). NND as a metric has been shown to lead to conclusions similar to those obtained using other similarity measures, such as dynamic time warping, the longest common subsequence, the edit distance for real sequences and the Fréchet distance, with a sensitivity only to a shifting point, or varying strength, of attraction (see [52]). Given that we do not expect the point or strength of attraction to be variable in our terns, which are highly site-faithful to both their breeding colony [41,42] and their non-breeding residence areas ([43] and see §3), and find the nature of NND very intuitive, we prefer using it over other metrics (for reviews, see e.g. [54,55]).

Since the non-breeding residence areas of individual common terns cover a very large latitudinal range ([43] and see §3), we restricted all tracks to the most northern wintering location (latitude: 21.15°N) to avoid overestimation of the NND when comparing the southern end of a longer focal track to a track with a more northerly wintering destination (see electronic supplementary material, figure S2b). Then, for each location of a track, we calculated the NND as the minimum Great Circle (orthodromic) distance to the nearest location(s) of a comparison track (see electronic supplementary material, figure S2a) with the R package ‘*geosphere*’ [56]. To ensure that differing numbers of locations per track and differing numbers of tracks per bird did not lead to certain tracks or individuals influencing our results more than others, we first calculated a mean NND for each pair of tracks (electronic supplementary material, figure S2a), then a mean NND for each bird and then a grand mean (i.e. the mean of means) from all within-individual bird means.

Once we had calculated a within- and among-individual grand mean NND, we sought to test whether these grand means were smaller than expected by chance, i.e. whether birds either showed within-individual spatial similarity in their migration routes across years or similar migration route selection with other birds leaving within ± 3 days of themselves. To do this, we compared our empirically observed grand mean NND to a ‘null’ estimate. This ‘null NND’ was calculated by comparing randomly selected pairs of tracks, sampled with replacement from the population as a whole. As described above, in each iteration of the randomization, we first calculated a mean NND for each track-to-track comparison, before calculating a mean per bird and a grand mean per iteration of the randomization. The randomization was repeated 1000 times, with each iteration representing a realistic, but ultimately random, NND. We then calculated the number of times the null NND value outperformed the true NND value (i.e. a *p*‐value). We corrected this *p*-value for the two-tailed nature of the test.

Although common terns show very little sexual dimorphism [57], females are known to leave the breeding colony roughly 2 weeks earlier than males [43]. As such, we repeated our analyses for each of the sexes to test whether the results obtained held for both males and females (electronic supplementary material, figure S3a), correcting the *p*-value for testing twice.

### (e) Among-individual temporal route similarity in response to wind

Birds have little control over the total head- or tailwind experienced during flight, since this is largely determined by environmental wind conditions, but can have more control over the proportion of the total wind vector that acts on them as headwind, since this can be changed by varying flight direction. To test whether the headwind proportion, i.e. the proportion of the total wind vector that is headwind (where −1 would be a complete tailwind and 1 would be a complete headwind), encountered by a bird was smaller when that bird was observed empirically than if it would have taken the same route at a different time, we first calculated the headwind experienced by each bird during its actual migration, i.e. excluding stationary periods (locations with a distance of <100 km from each other which we assumed to be a stopover, as common terns generally migrate much further between two twilights). Given that terns mostly fly at low altitude, following a fly-and-forage migratory strategy [58], we used wind data at 10 m from the European Centre for Medium-Range Weather Forecasts Weather Reanalysis 5 (ERA5 10 m wind [59], downloaded in 2022 from https://cds.climate.copernicus.eu). To calculate the headwind component experienced by the bird, we multiplied the cosine of the angle between the wind direction and the bird’s direction of travel by wind speed computed for successive locations [27]. From this, we calculated a headwind proportion as the headwind component divided by the total wind speed (similar to [37]). Once a headwind proportion, therefore, was calculated per location, we calculated a mean headwind proportion per track and, in turn, per bird, from which we calculated a grand mean. We then randomized the departure date by assigning birds a departure date selected at random from every observed departure date (with each departure date represented proportionally to the distribution of departure dates to avoid bias) and re-calculated the headwind proportion per track, then per bird, to estimate the grand mean headwind proportion and test whether the observed headwind proportion was smaller than expected by chance. We repeated this step 10 000 times before we again calculated the proportion of iterations where the null model outperformed the observed data (hence determining the *p*‐value). In our randomization procedure, we did not restrict assignments of departure date within seasons, because we were only able to track relatively few birds each season, such that we would only be able to randomize across a rather limited range of environmental variation. Given that our question pertained to the overall potential wind conditions a bird could have potentially experienced, our aim was to include as wide a range of wind conditions at which terns were observed to migrate (i.e. not forego migration because conditions were too poor to fly) as possible.

## 3. Results

**Figure 1.**
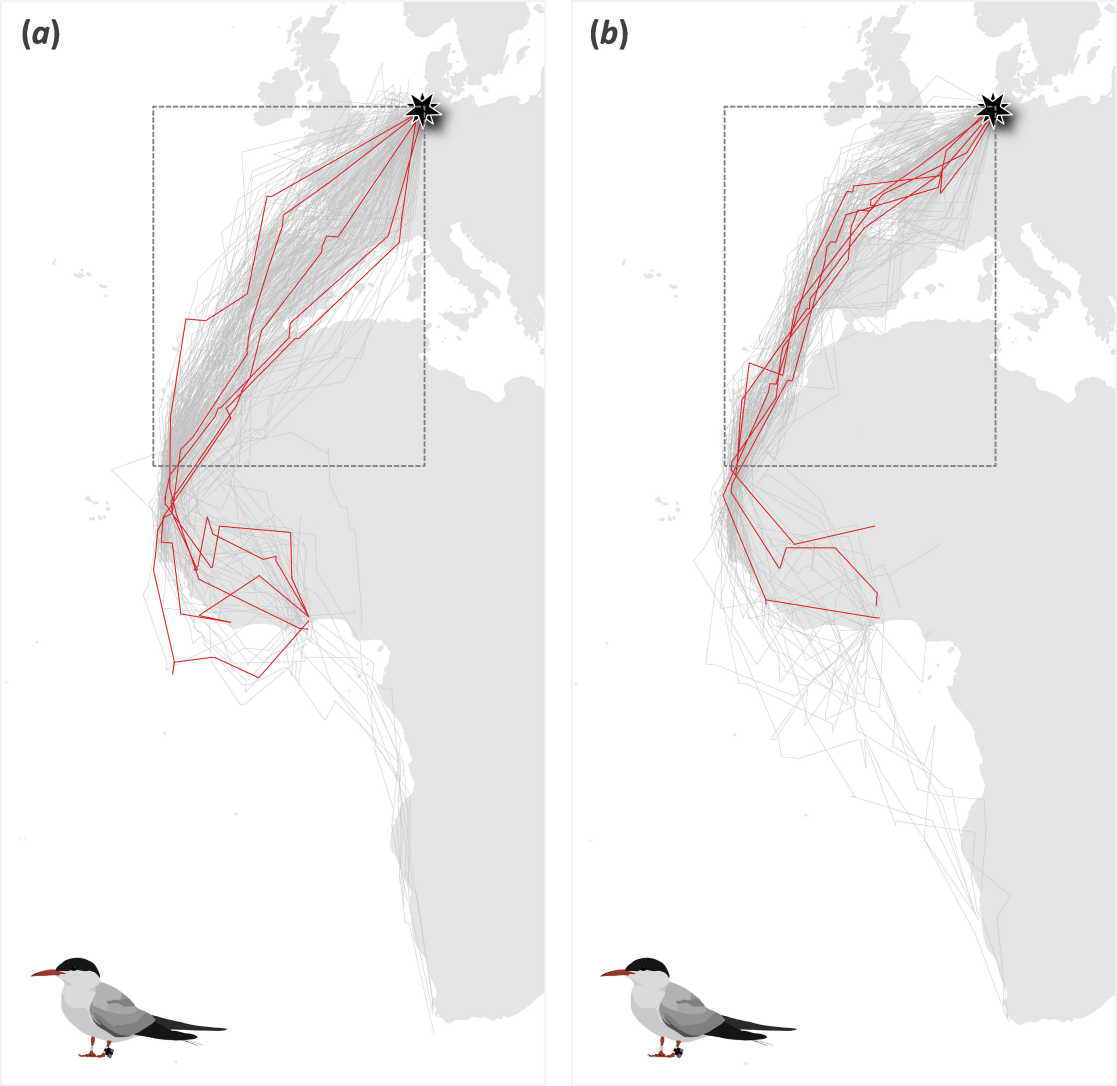
(*a*) Little within-individual similarity in seasonal migration routes across years: an example of route dissimilarity, with a single common tern travelling the five post-breeding migration routes highlighted in red, with the full set of post-breeding migrations of the tracked population in grey. (*b*) Stronger among-individual temporal similarity in seasonal migration routes: an example of route similarity, with five common terns travelling similar pre-breeding migration routes (highlighted in red) when leaving the breeding colony within the same time window (± 3 days), with the full set of pre-breeding migrations of the tracked population in grey. Analyses were based on the parts of the tracks shown in the box, i.e. between the breeding colony (53.51°N) and the most northern non-breeding residence area (21.15°N; see §2).

Across 192 post-breeding migratory journeys made by 84 individuals, common terns on average left the breeding colony on 7 September, arriving at their non-breeding residence area on 20 September. The individual repeatability of their departure and arrival date was substantial: 0.65 ± 0.06 (CI = 0.58, 0.83; *p* < 0.001) and 0.57 ± 0.07 (CI = 0.43, 0.70; *p* < 0.001), respectively. Non-breeding residence areas were distributed along the coast of Mauretania, Senegal, The Gambia, Guinea Bissau, Guinea, Sierra Leone, Ghana, Nigeria, Namibia and South Africa (figure 1), and the individual repeatability of the longitude and latitude of these non-breeding sites was very high: 0.99 ± ≤0.00 (CI = 0.98, 0.99; *p* < 0.001) and 0.93 ± 0.02 (CI = 0.90, 0.95; *p* < 0.001), respectively. Across 149 pre-breeding migratory journeys made by 75 of the individuals, common terns left the non-breeding residence area on average on 1 April, arriving at the breeding colony on 22 April. The individual repeatability of the departure date was moderate (0.38 ± 0.10; CI = 0.19, 0.57; *p* < 0.001), while that of the arrival date again was substantial (0.70 ± 0.60; CI = 0.58, 0.81; *p* < 0.001).

**Figure 2.**
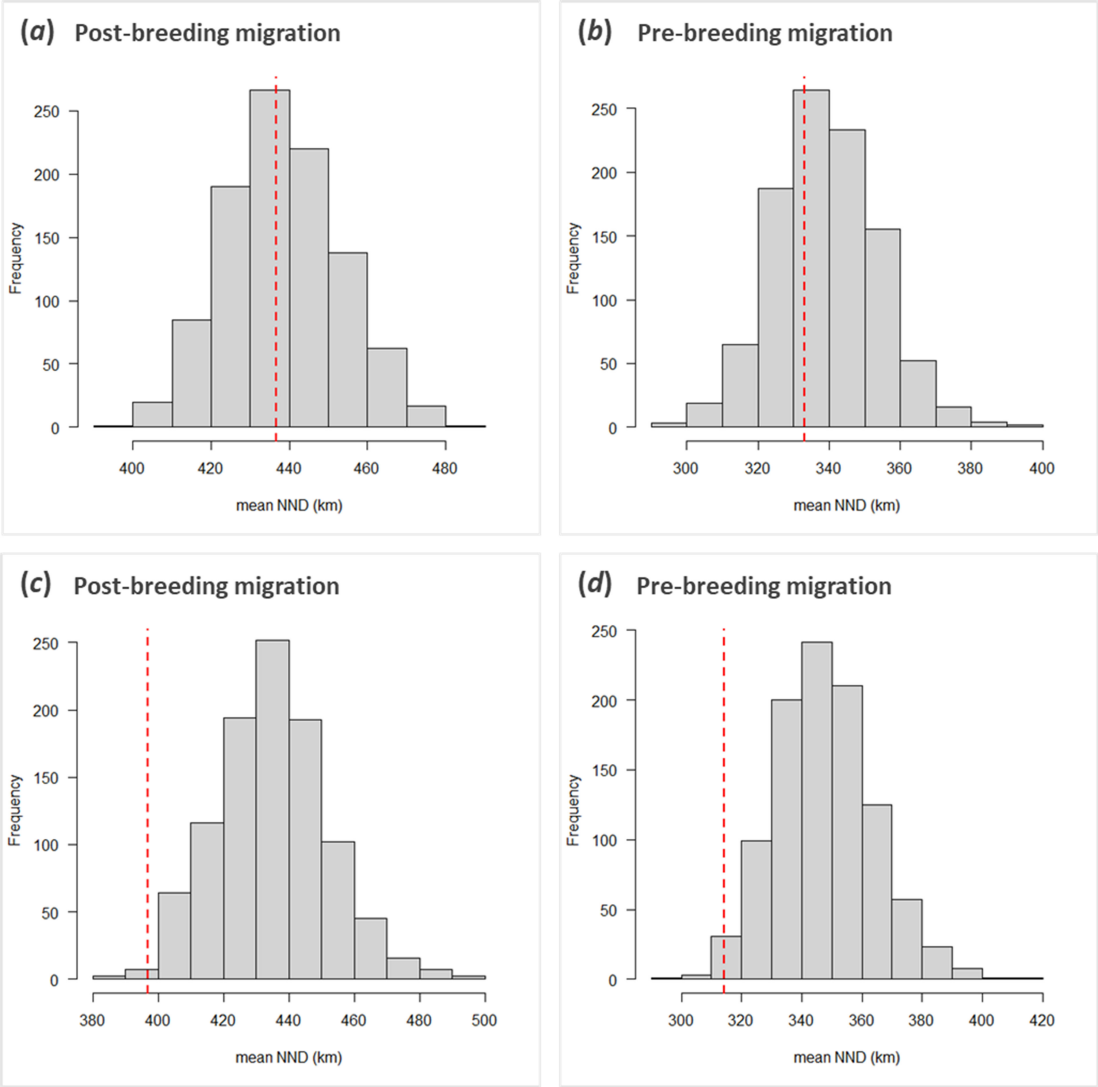
(*a* and *b*) Little within-individual similarity in seasonal migration routes across years: frequency histograms of the randomized nearest neighbour distance (NND) compared with the observed within-individual NND (dotted red line) during post-breeding and pre-breeding migration. (*c* and *d*) Stronger among-individual temporal similarity in seasonal migration routes: frequency histograms of the randomized NND compared with the observed temporal NND (dotted red line) during post-breeding and pre-breeding migration.

With respect to the routes taken, the average (±s.e.) NND was 437 ± 20 km during post-breeding migration and 333 ± 20 km during pre-breeding migration. We did not find within-individual similarity across years either during post-breeding (randomization analysis; *p* = 0.940; figures 1*a* and 2*a*) or pre-breeding (randomization analysis: *p* = 0.712; figure 2*b*) migration. The same held true for both sexes when analysing the tracks of males and females separately, although for pre-breeding migration, there was a tendency for females to take a somewhat more, and for males to take a somewhat less, similar route to themselves than to randomly selected other birds (pre-breeding randomization: *p* = 0.216 (females) and *p* = 0.060 (males); electronic supplementary material, figure S3). This was not the case during post-breeding migration when the effects were very similar between, and clearly non-significant for, both sexes (post-breeding randomization: *p* > 0.999 (females) and *p* > 0.999 (males); electronic supplementary material, figure S3).

**Figure 3.**
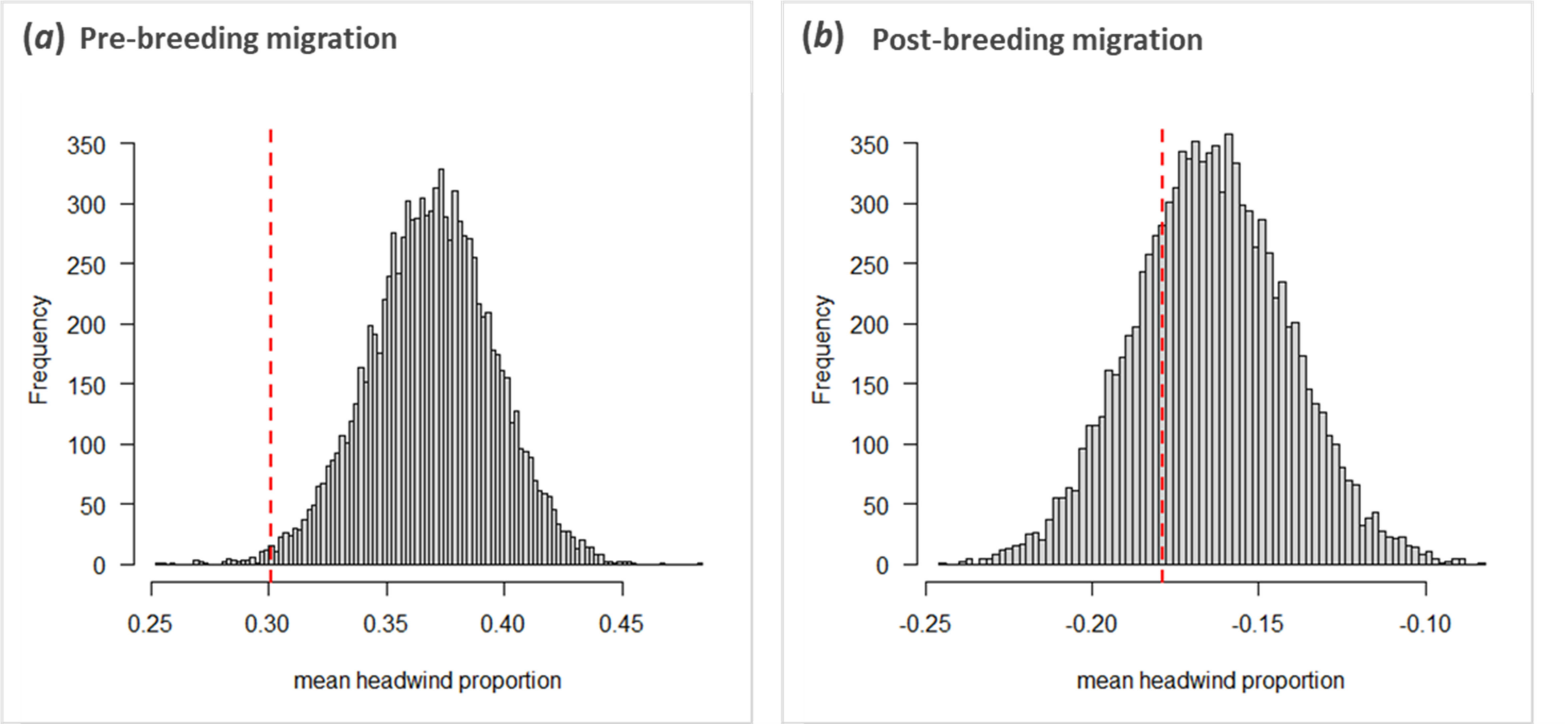
Among-individual similarity in seasonal migration routes of birds leaving within the same time window (‘temporal similarity’) is associated with headwind minimization during pre-breeding migration. Frequency histograms of the randomized headwind (positive values) or tailwind (negative values) proportions (calculated by dividing the headwind component by the total wind speed, computed for successive locations) individuals would have encountered should they have taken the same route, but have left at a weighted random other date than they did, compared with that they were observed to have encountered (scattered red line) during (*a*) pre-breeding and (*b*) post-breeding migration.

Instead, we found birds leaving within the same time window (± 3 days) to travel along more similar routes than expected by chance, both during post-breeding (randomization analysis: *p* = 0.012; figure 2*c*) and pre-breeding (randomization analysis: *p* = 0.030; figures 1*b* and 2*d*) migration. During pre-breeding migration, the proportion of headwind birds experienced using their route on their actual day of departure (observed proportion of headwind: 0.30) was lower than the headwind they would have experienced should they have used that route on a random day on which one of their peers departed for migration (range of the headwind proportion for the observed route when it would have been executed at any other observed departure date: 0.25–0.48; randomization analysis: *p* = 0.012; figure 3*a*). This was not the case during post-breeding migration (randomization analysis: *p* = 0.535; figure 3*b*), most likely because the birds encountered more tailwinds when heading south (observed proportion of tailwind: −0.18; range of the tailwind proportion for the observed route when it would have been executed at any other observed departure date: −0.24 to −0.08, i.e. all negative). Although the observation that birds encounter more tailwinds across their southward migration might initially seem unintuitive, since winds in Europe come predominantly from the southwest [60], a considerable proportion of the route is undertaken across northern Africa with support from the northern trade winds. This leads to an average wind support on the southbound leg, which might explain why wind selectivity was more prevalent on the northbound leg.

## 4. Discussion

To increase our understanding of individual and environmental sources underlying variation in migration routes, we analysed repeated tracks obtained from a long-distance migratory seabird, the common tern. These analyses indicate that these birds show little within-individual similarity in their season-specific migration routes taken across years, but instead show substantial among-individual temporal route similarity, meaning that birds departing within the same time window (± 3 days) take similar routes. During pre-breeding migration, this among-individual temporal route similarity was associated with an effective minimization of the headwind proportion encountered. During post-breeding migration, birds were found to experience tailwind, such that we expect their temporal route similarity to be underpinned by optimization with respect to other, yet unidentified, environmental variables.

Since motile animals, such as birds, must cope with a highly dynamic and complex environment (e.g. [31]) and trade-offs limit flexibility, it follows that their behaviour cannot be optimized with respect to all environmental factors encountered. High spatial consistency in the migration route, for example, would result in low consistency with respect to the environment the birds migrate through. What birds optimize must, then, either reflect an individual’s priorities or, alternatively, limitations in the mechanisms that underpin migration. Our finding that individual common terns flexibly take routes that effectively minimize the headwind they experience during pre-breeding migration, and perhaps thereby show substantial among-individual temporal route similarity, suggests that wind selection is likely an essential process by which these birds migrate most efficiently (at least in situations in which encounters with headwind are plentiful). This can be explained by the increase in flight costs experienced when flying in a strong headwind (e.g. [38,61,62]) and is consistent with results from previous studies across both sea (e.g. [34,37,63–66]) and land birds (e.g. [67–74]). Indeed, almost all studies assessing optimal route-following in a fluid medium suggest that drifting at least partially with the wind is adaptive during directed flight since this allows the maximization of overall energetic efficiency while maintaining an approximately goalward course (e.g. [75,76]). This, however, is likely to require quite complex cognitive skills, not only because birds must gauge the cost/benefit of different routes, but also because they must be capable of flexibly determining their displacement from the goal on a near-instantaneous basis. This, in turn, would likely require a mechanism to either determine position relative to the goal (i.e. a cognitive ‘map’; e.g. [22,23,27,77]) or a highly accurate mechanism of keeping track of the outward journey (i.e. a path integrator; e.g. [78–80]).

Interestingly, the limited encounter of headwind during post-breeding migration did not lead to birds being consistent in their route choice during this part of the annual cycle. Instead, the average distance birds had to their own tracks was even a little larger during post- compared with pre-breeding migration. Given that the proportion of tailwind experienced was not significantly maximized either, we speculate that post-reproductive birds experiencing benign wind conditions for migration optimize their route to a currently unidentified environmental variable. With common terns using a fly-and-forage migratory strategy (e.g. [58]), and having been reproductively active is likely to come with a loss of energy stores [81], this environmental variable may be more closely related to food availability. It should be noted, however, that if so, this foraging behaviour is not very time-consuming, since post-breeding migration is faster and more navigationally efficient than pre-breeding migration [82].

Overall, our study adds to the relatively small body of work showing that individuals use flexible migration routes across years (e.g. [12–19], but see [20]). It suggests that within-individual changes allow for a potentially adaptive behavioural response to environmental conditions [83], which we here suggest to be wind direction and speed (for a review, also see [39]). In addition to highlighting the importance of longitudinally collecting tracking data when evaluating the drivers of spatial variation in long-distance migration, our study raises questions regarding the fitness consequences and mechanistic underpinning of the birds’ ability to flexibly adjust routes to stochastic environmental variation.

## Data Availability

The R code for the analysis of our light-level geolocator data is provided in [43]. The R code and the corresponding data for the NND and wind analyses are available on Dryad [84]. Supplementary material is available online [85].
